# Correction: Integrated water quality dynamics in Wadi Hanifah: Physical, chemical, and biological perspectives

**DOI:** 10.1371/journal.pone.0336505

**Published:** 2025-11-07

**Authors:** Hazem Aqel, Naif Sannan, Afnan Al-Hunaiti, Ramy Fodah

There is an error in affiliation 1 for author Hazem Aqel. The correct affiliation 1 is: Basic Medical Sciences Department, College of Medicine, Al-Balqa Applied University, Salt, Jordan.
