# Supplementary material for: Integrated water quality dynamics in Wadi Hanifah: Physical, chemical, and biological perspectives
Source: PLoS One. 2024 Feb 15;19(2):e0298200. doi: 10.1371/journal.pone.0298200 (PMC10868741; doi:10.1371/journal.pone.0298200)

**Supporting information**

**S1 Fig.** **Natural water source’s location in Wadi Hanifah. L1-L10: Water sample before the filtration and water treatment; L11: Water sample after entering station door with simple filter; L12: Water sample after first water treatment; L13: Water sample after second water treatment, using filter, Fish and algae; and L14: Water sample after final filtration.**


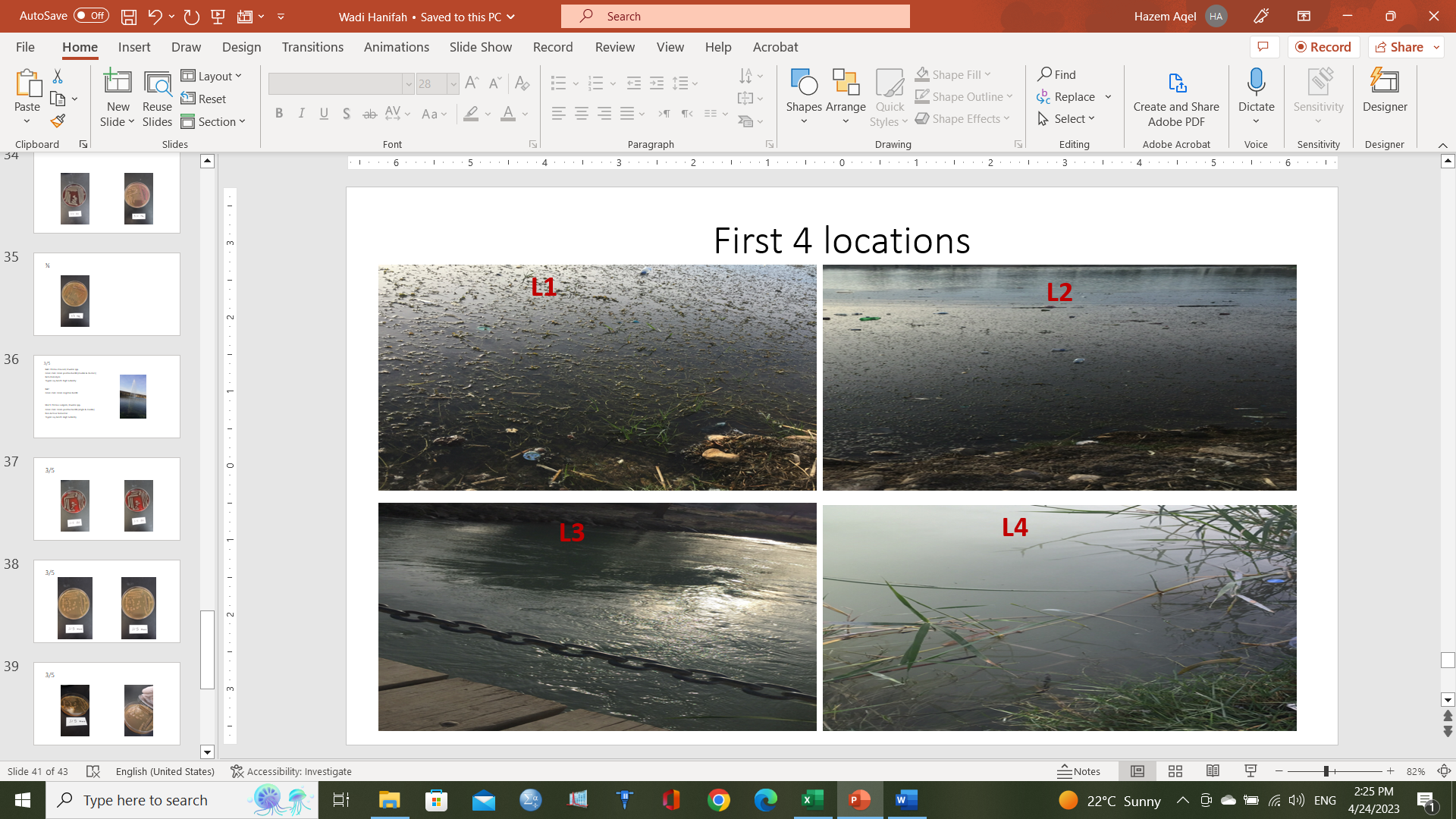


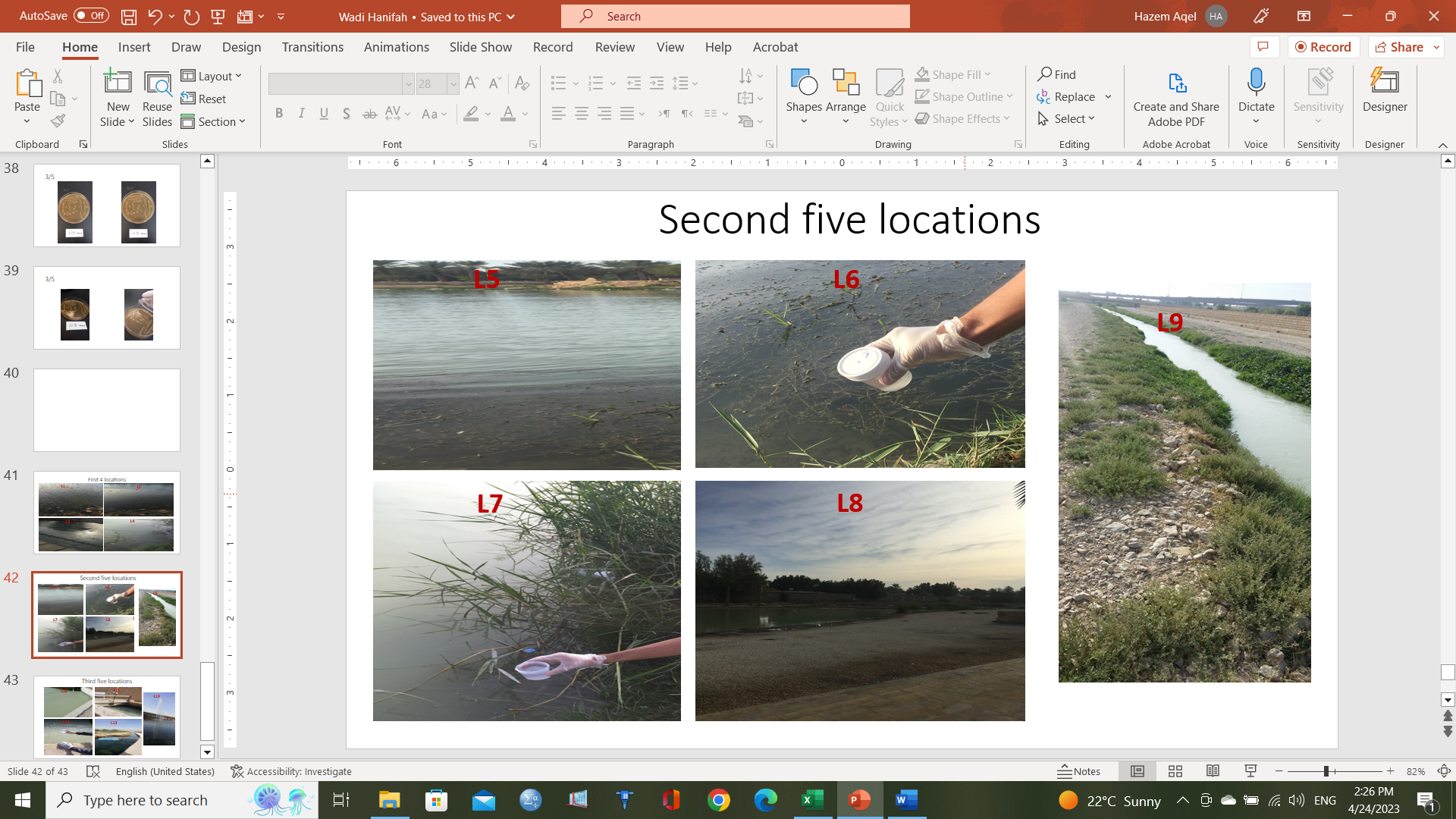


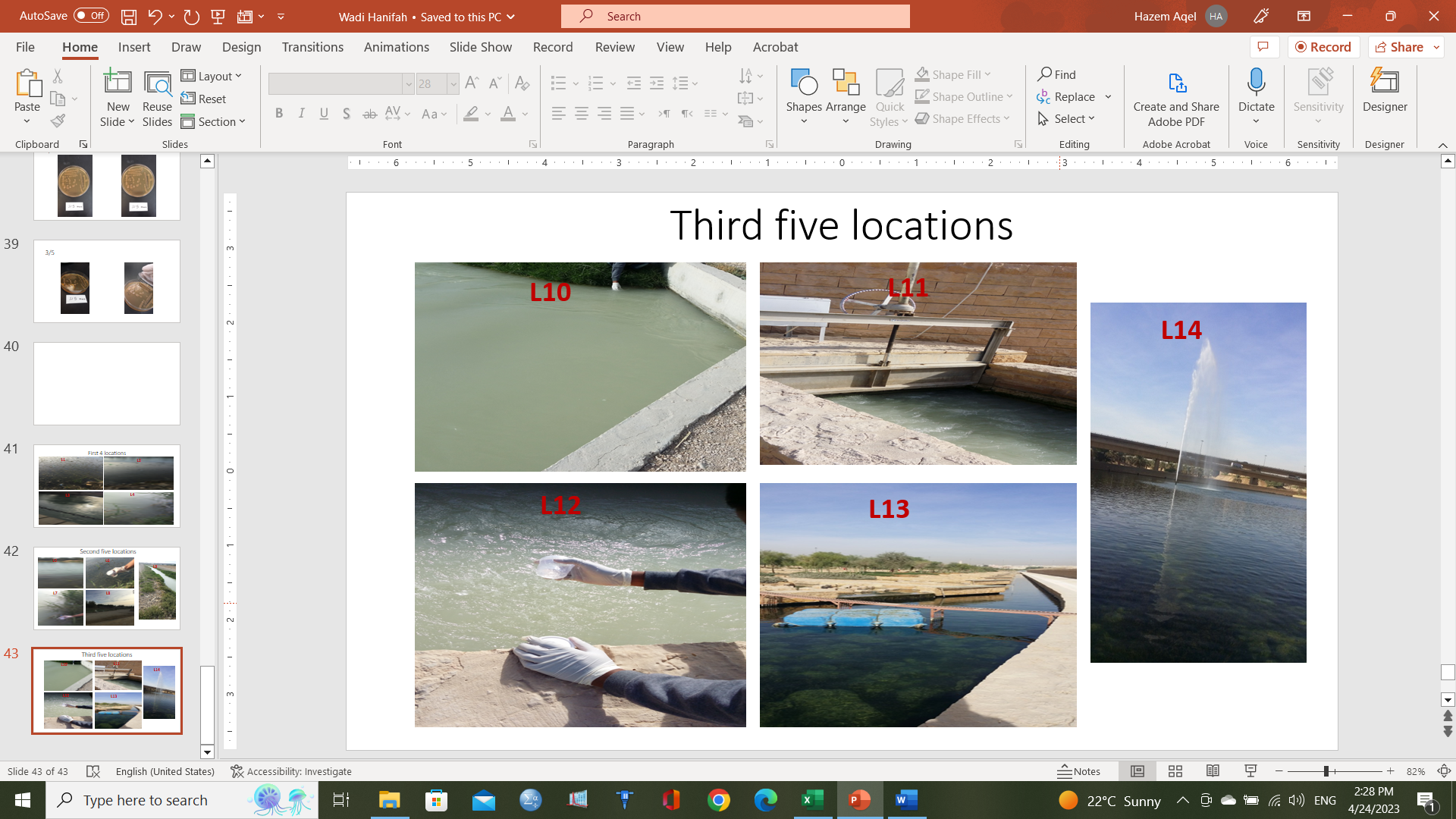

Supplement: S1 Fig — L1-L10: Water sample before the filtration and water treatment; L11: Water sample after entering station door with simple filter; L12: Water sample after first water treatment; L13: Water sample after second water treatment, using filter, Fish and algae; and L14: Water sample after final filtration. (DOCX) [file pone.0298200.s001.docx]
