# Supplementary material for: Integrated water quality dynamics in Wadi Hanifah: Physical, chemical, and biological perspectives
Source: PLoS One. 2024 Feb 15;19(2):e0298200. doi: 10.1371/journal.pone.0298200 (PMC10868741; doi:10.1371/journal.pone.0298200)
Supplement: Attachment — Submitted filename: Response to Reviewer Comments.docx [file pone.0298200.s003.docx]

**Response to Reviewer Comments**

We appreciate the valuable comments and suggestions provided by the reviewer. Below, we address each comment and describe the revisions made to the manuscript accordingly.

**Introduction**

1. **Expansion on the history and importance of Wadi Hanifah:** We have expanded the introduction to include more details about the history and significance of Wadi Hanifa (Lines 62-69).
2. **Differentiation from previous studies:** A detailed comparison with previous investigations has been added, highlighting how this study differs from other studies. This comparison is presented in paragraphs rather than a table for narrative clarity (Lines 70-99).

**Materials and Methods**

1. **Study area-selection of locations:** The rationale for selecting the 14 locations for the study has been clearly articulated in the revised manuscript (Lines 128-133).
2. **Collection of water samples:**

- Corrected the reference to Figure 2, now labeled as S2 Fig (Lines 136-137).
- Information about the duration of sample collection from all locations is now included (Lines 140-142).

3. **Assessment of physicochemical characteristics:**

- Details about the laboratory instruments and principles used for measurements are now provided (Lines 150-164).
- The method of sample transportation to the laboratory is explained (Lines 170-173).
- Clarification is added regarding the timing of sample analysis upon receipt (Lines 174-176).
- The testing site for physicochemical measurement is specified as KAIMRC Laboratories.
- A reference for the American Public Health Association guidelines is now included (Line 158).
- Additional details and references for the standardized methods used to measure nitrite, nitrate, ammonia, and phosphate levels are provided (Lines 158-160).

**Results (Results and Discussion combined)**

1. **Physical characteristics of wadi Hanifah:**

- Methodology for odor testing is explained (Line 146-149).
- Justifications for grouping some locations in Table 1 are included (Lines 245-246).
- Details about heavy metal analysis instruments are now added (Lines 165-169).
- Values in Table 2 are color-coded to indicate their relation to standard values (Red: above; Green: Equal; Blue: Below).

**Discussion**

1. **Temperature range and aquatic life:** Elaboration on how the temperature range of 23.2°C to 27.4°C affects aquatic life and chemical reactions is provided (Lines 206-214).
2. **Details about total dissolved solids (TDS):** Additional information about TDS is included (Lines 274-284).
3. **Management and responsibility:** Clarifications regarding who should take responsibility and what actions are suggested based on the study outcomes are addressed (Lines 378-383).

We believe these revisions adequately address the concerns raised by the reviewer and enhance the quality and clarity of the manuscript. We appreciate the opportunity to improve our work based on these insightful comments.
