# Supplementary material for: Integrated water quality dynamics in Wadi Hanifah: Physical, chemical, and biological perspectives
Source: PLoS One. 2024 Feb 15;19(2):e0298200. doi: 10.1371/journal.pone.0298200 (PMC10868741; doi:10.1371/journal.pone.0298200)
Supplement: Attachment — Submitted filename: reviewer comments.pdf [file pone.0298200.s002.pdf]

Editor in Chief, PLOS ONE

Integrated Water Quality Dynamics in Wadi Hanifah: Physical, Chemical, and Biological Perspectives

PONE-D-23-42265

Reviewer(s)' Comments to Author:

Reviewer:

Comments to the Author

The manuscript describes comprehensive evaluation of water quality in one of the known valleys in Arabian Peninsula of Saudi Arabia. Analysis included physical and Chemical constituents and bacterial contamination.

Specific comments

Manuscript:

Introduction

- The authors need to expand in more detail about the history & importance of Wadi Hanifa.
- How this study about Wadi Hanifa will be different from previous investigations about Wadi Hanifa (Ref. 15,16). This can be presented in a short literature using a table of summary.

Material and methods

Study area

- Not enough information was provided to show the reason for the selected 14 locations?

Collection of water samples

- Line 89: Figure 2 cannot be found.
- How many days were taken to complete the samples collection from all locations?

Assessment of the physicochemical characteristics

Physicochemical characteristics measured to determine total alkalinity, hardness, calcium, magnesium, sulfate nitrate, ammonia, and phosphate levels.

- No laboratory instruments and principles details were provided.
- How were samples transported to the laboratory for measurement?
- Indicate if samples analysis were performed immediately upon receiving or performed as batching (all together).
- After collecting the samples, where was the testing site performed for physiochemical measurement? e.g laboratories of Water Ministry, Ministry of Agriculture, University, etc
- Line 97: reference needs to be cited for the American Public Health Association guidelines.

- Line 98: Nitrite, nitrate, ammonia, and phosphate levels were measured using standardized methods. More details with references are required for standardized methods.
- Figure 1: figure's resolution not so clear, no indication for Wadi Hanifa limits or borders, many key numbers included (e.g. 500, 535 etc.) but no keywords.

Microbial cultivation and identification

- Line 105: Bacterial strains were isolated from the collected water samples using various of culture media. What kind of media were used? Where was the sample culture performed?

Results:

- It has been noticed that detailed results justifications were discussed in the section of results. In the section of results as the main results and outcomes can be described only but without further discussion as further discussion can be mentioned in the section of discussion.

Physical characteristics of Wadi Hanifah

- Line 135: how odor testing was performed?
- Table 1: some locations were joined together such as L1-L4, L5-L10, L11-L12, were there any justification to give the same exact readings?
- Line 161: The heavy metal analysis for Manganese, iron, and chromium levels were performed but no instrumental details were mentioned in the section of methods and materials.
- Table 2: the values need to be color coded for > or < the standard values.
- Table 2: Reference or references for the standard values need to be cited.

Discussion:

- Line 322 -323: how can a range of temperature from 23.2°C to 27.4C be associated with aquatic life and chemical reaction?
- Line 340: more details about TDS are required.
- In the discussion and conclusion, the authors mentioned that "more attention and management" are required but the authors did not mention required exactly from whom and who should be responsible for such action to take and what suggestions can be provided by the authors to the responsible body according to study outcomes.
